# Uncovering SOD3 and GPX4 as new targets of Benzo[α]pyrene-induced hepatotoxicity through Metabolomics and Chemical Proteomics

**DOI:** 10.1016/j.redox.2023.102930

**Published:** 2023-10-11

**Authors:** Yanwei Wang, Jiahui Zhao, Yipeng Xu, Cimin Tao, Jie Tong, Yingjie Luo, Yong Chen, Xuesong Liu, Tengfei Xu

**Affiliations:** a Key Laboratory of Advanced Drug Delivery Systems of Zhejiang Province, College of Pharmaceutical Sciences, Zhejiang University, Hangzhou, 310058, Zhejiang, China; b Department of Geriatrics and Shenzhen Clinical Research Centre for Geriatrics, Shenzhen People’s Hospital (The Second Clinical Medical College, Jinan University, The First Affiliated Hospital, Southern University of Science and Technology), Shenzhen, Guangdong, 518020, China; c Department of Urology, Zhejiang Cancer Hospital, Hangzhou, Zhejiang, 310022, China; d PET Center, Department of Radiology and Biomedical Imaging, Yale School of Medicine, New Haven, CT, 06520, USA; e Cangnan County Qiushi Innovation Research Institute of Traditional Chinese Medicine, Wenzhou, Zhejiang, 325899, China

**Keywords:** Benzo[α]pyrene, Superoxide dismutase 3, Glutathione peroxidase 4, Metabolomics, Chemical proteomics

## Abstract

Benzo[α]pyrene (Bap) is recognized as a ubiquitous environmental pollutant among the polycyclic aromatic hydrocarbons (PAHs) class. Previous studies have shown that the hepatotoxicity of Bap is mainly caused by its metabolites, although it remains unclear whether Bap itself induces such damage. This study integrated metabolomics and chemical proteomics approaches to comprehensively identify the potential target proteins affected by Bap in liver cells. The results from the metabolomics showed that the significant changed metabolites were related with cellular redox homeostasis. CEllular Thermal Shift Assay (CETSA) showed that Bap induced protein thermal displacement of superoxide dismutase 3 (SOD3) and glutathione peroxidase 4 (GPX4), which are closely related to oxidative homeostasis. Further validation through *in vitro* CETSA and drug affinity response target stability (DARTS) revealed that Bap directly affected the stability of SOD3 and GPX4 proteins. The binding affinities of Bap to the potential target proteins were further evaluated using molecular docking, while the isothermal titration calorimetry (ITC) interaction measurements indicated nanomolar-level Kd values. Importantly, we found that Bap weakened the antioxidant capacity by destroying the activities of SOD3 and GPX4, which provided a new understanding of the mechanism of hepatotoxicity induced by Bap. Moreover, our provided workflow integrating metabolomics and label-free chemical proteomics, can be regarded as a practical way to identify the targets and inter-mechanisms for the various environmental compounds.

## Highlights

• Benzo[α]pyrene induced oxidative damage by disrupting the antioxidant system of liver cells.
• Benzo[α]pyrene had a strong affinity with SOD3 and GPX4.
• Benzo[α]pyrene significantly reduced the activities of SOD3 and GPX4.
• Propose a method for screening environmental pollutant targets by combining metabolomics with Cellular Thermal Shift Assay.

## 1 Introduction

With the development of industrialization, environmental pollutants polycyclic aromatic hydrocarbons (PAHs) have received more and more attention. Especially in areas with frequent industrial activities, polycyclic aromatic hydrocarbons emissions are significantly higher [1,2]. Benzo[α]pyrene (Bap) as a highly toxic polycyclic aromatic hydrocarbons subtype, Bap is often used as an indicator to characterize the total load of polycyclic aromatic hydrocarbons in a given environment [3]. Substantial kinds of literature indicate that Bap tends to accumulate within the body following exposure [4,5]. A study found that 462 subjects exposed to Bap had an average blood concentration of 140 ng/mL of Bap in their blood [5], while mice exposed to 125 mg/kg/d of Bap for one month had an accumulation of 150 ng/mL of Bap in the blood [6]. In addition, Bap has a strong carcinogenic toxicity, so it is listed as the first category” human carcinogen” by the World Health Organization (WHO) International Agency for Research on Cancer [7].

Previous studies have shown that the toxic effects of Bap are mainly mediated by reactive intermediates produced through multiple or continuous metabolic transformations [8]. Initially, Bap was metabolized by cytochrome P450 (CYP) enzymes to epoxides, which were then hydrated by epoxide hydrolases to various dihydroglycols. Among them, Bap-7,8-dihydrodiol 9,10-epoxide (BPDE) produced by the further oxidation of *trans*-7,8-dihydroxy-7,8-dihydro-bep can covalently combine with DNA to form BPDE-DNA adduct, leading to DNA mutation [9,10]. Therefore, aromatic hydrocarbon receptor (AhR) activators, such as 2,3,7,8-tetrachlorodibenzo (p) dioxins, may increase Bap toxicity by inducing CYP1A1 [11]. In addition, Bap also forms phenols through various pathways, such as spontaneous rearrangement of epoxides. These phenols can be converted into quinones and produce reactive oxygen species through redox cycles [12]. The oxidative effects of Bap are primarily attributed to its metabolites, particularly BPDE, and ROS generated during the transformation process [13,14]. Although the liver is particularly vulnerable to damage, it is not yet clear whether Bap itself can induce hepatotoxicity [15,16]. Identifying the target proteins of Bap during metabolic regulation would represent a significant advancement in knowledge.

The identification of proteins with direct binding capacity to Bap is of utmost significance, as they represent essential enzymes or regulatory proteins influencing toxicity. Various chemical proteomics techniques have been developed, such as drug affinity response target stability (DARTS) [17], activity-based protein profiling (ABPP) [18], protein chip [19] and stability of proteins from rates of oxidation (SPROX) [20]. However, most of these methods require specific treatment of compounds, which may alter their chemical structures and properties, rendering them unsuitable for screening of certain compounds on a high-throughput basis. Bap, with its inflexible rigid structure of several benzene rings connected together, does not carry heteroatoms or modifiable groups, necessitates the use of label-free chemical proteomics to screen for potential targets. The CEllular Thermal Shift Assay (CETSA) method is capable of detecting intracellular compounds binding efficiency with target proteins without any special treatment. Its principle is that the thermal stability of target proteins undergoes changes following binding to the ligands [21]. Our previous studies utilized CETSA to investigate the mechanisms of MEHP leading to cellular fat accumulation [22] and cytotoxicity [23], monitor ROS and redox modulations of protein structure at the proteome level [24], validating it as a reliable method to screen environmental pollutant targets and protein redox modulations.

The present study utilized metabolomics to comprehensively characterize metabolic alterations in hepatic cells upon Bap treatment, and further screened the Bap interacting proteins using the CETSA method. By integrating the two platforms, active ligand-binding proteins could be identified using a “bottom-up” approach. This integration led to the discovery of SOD3 and GPX4 as being Bap active proteins, thereby providing novel insights into the potential mechanisms of Bap-induced hepatotoxicity.

## 2 Materials and methods

### 2.1 Experimental workflow

**Fig. 1:**
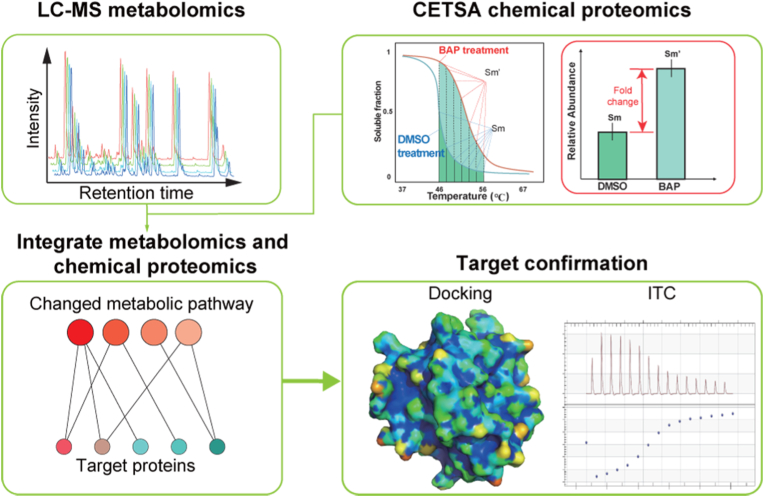
Schematic diagram of Bap-targeted proteins screening. In the proposed method, metabolomics was used to characterize the metabolome changes after Bap exposure. Chemical proteomics was further used to screen out the target proteins whose thermal stability was changed after Bap exposure, while related to the altered metabolites affected by Bap. Molecular docking and isothermal titration calorimetry (ITC) further confirmed the interaction between Bap and the selected target proteins.

As shown in Fig. 1, this study combined metabolomics and chemical proteomics to screen target proteins of Bap. Firstly, metabolomics was used to screen metabolites that changed after Bap exposure. Secondly, CETSA chemical proteomics mapped the target proteomes of Bap near the median of protein thermal melting temperature. Afterward, the enriched metabolomics pathway was associated with the target proteins screened by CETSA. Finally, the affinity between Bap and potential new targets was analyzed using molecular docking and ITC interactions.

### 2.2 Selecting Bap exposure concentration for metabolomics

Mouse hepatocellular cell line (AML12) acquired from American Type Cell Culture (ATCC, Manassas, VA) was employed as the *in vitro* model. The cells were seeded in 96-well plates at a density of 5 × 10^4^ cells mL^−1^ and maintained at 37 °C with 5% CO_2_ in Dulbecco's Modified Eagle Medium containing 10% fetal bovine serum, 2 mM l-glutamine and 1% pyruvate. The cell viability experiment was conducted with various concentrations (0.0064, 0.032, 0.16, 0.8, 4, 20, 100 and 500 μM) of Bap (with 0.5% DMSO) after 24 h incubation by adding Cell Counting Kit - 8 (CCK8) Reagents (Thermo Scientific, Singapore), and reading the absorbance at 450 nm according to the product instruction. For metabolomics analysis, the selection of Bap dosages was referred to previous studies [[25], [26], [27]], the cells were treated with Bap at concentrations of 0.8, 4, and 20 μM (with 0.5% DMSO) for 24 h, the vehicle controls were incubated with 0.5% DMSO. All the groups have three biological replicates.

### 2.3 Metabolomics analysis

The specific steps for metabolite extraction, analysis, and identification refer to Text S1 in the supporting information.

### 2.4 Determination of GSH and GSSG levels

GSH and GSSG were analyzed by Agilent 6545 Q-TOF liquid chromatography-mass spectrometry (Agilent, America) equipped with a 1290 Infinity II liquid chromatography system and 6545 time of flight (TOF/Q-TOF) high-resolution mass spectrometry using a using Agilent InfinityLab Poroshell 120 EC-C18 Column (2.1*50 mm, 1.9- μm) as described previously [28].

### 2.5 Relative quantification of reactive oxygen species (ROS)

Total ROS levels were assessed in the cells using a Reactive Oxygen Species Assay Kit (Beyotime, China) in accordance with the manufacturer's instructions. Briefly, the cells were processed with 0, 0.8, 4, 20 μM Bap treated AML12 cells for 1 h and 24 h, and then 10 μM DCFH-DA diluted in serum-free culture medium was added to the cells and incubated at 37 °C for 20 min. The cells were subsequently washed three times with serum-free cell culture medium to remove excess DCFH-DA. The fluorescence of DCF was analyzed using a microplate reader. Relative quantification of ROS levels was performed based on the cell viability.

### 2.6 Determination of 8-OHDG and MDA levels

8-OHDG was analyzed by LC/MS using an Agilent 6545 Q-TOF liquid chromatography-mass spectrometry (Agilent, America) equipped with a 1290 Infinity II liquid chromatography system and 6545 time of flight (TOF/Q-TOF) high-resolution mass spectrometry using a waters acquity uplc beh amide column (2.1*100 mm,1.7 μm) as described previously [29]. MDA was analyzed by LC/MS using an Agilent 6545 Q-TOF liquid chromatography-mass spectrometry (Agilent, America) equipped with a 1290 Infinity II liquid chromatography system and 6545 time of flight (TOF/Q-TOF) high-resolution mass spectrometry using a waters acquity uplc beh amide column (2.1*100 mm,1.7 μm) as described previously [30].

### 2.7 CETSA

AML12 cells were exposed to the complete culture medium containing 0.5% DMSO, 4, and 100 μM Bap, incubated at 37 °C in a 5% CO_2_ incubator for 1 h. Subsequently, cells were washed twice by PBS, and the cell suspensions were collected and centrifuged at 300×*g* for 5 min. The cell pellets were collected and resuspended in a PCR tube with 30 μL PBS. Divided each group of samples into 7 parts and heated them at 37, 46, 48, 50, 52, 54, and 56 °C for 3 min. Subsequently, merged the same group of samples and added lysis buffer (50 mM HEPES, pH 7.5, 0.1 mM activated Na_3_VO_4_, 5 mM β- Glycerophosphoric acid, 10 mM MgCl_2_, 1 mM TCEP and 0.1% EDTA free protease inhibitor), freeze-thaw repeatedly for 4 times with liquid nitrogen, then centrifuged to obtain the supernatant. The process of protein reduction, alkylation, digestion, TMT labeling, and analysis was based on previous research [22], and the specific steps refer to Text S2 in the supporting information.

### 2.8 CETSA data processing

Information of peptide and reporter ions of the TMT was extracted from Proteome Discoverer 2.1. The quantification of the binding proteins was based on the unique peptide intensities. The proteins with fold change greater than 1.2 in either 4 and 100 μM treatment group were screened as Bap's potential targets.

### 2.9 Western blotting

The detail western blotting steps were referred to previous literature [31].

(1) Western blotting for CETSA verification: Proteins were extracted from the cell lysate without phenyl methane sulfonyl fluoride (PMSF), and the protein concentration was measured using BCA. The cell lysate was treated with 0.5% DMSO, 4 and 100 μM Bap for 10 min. Divided each group of proteins into three tips, heated them in metal baths at 37, 48, and 56 °C for 3 min, and then transferred to the ice for protein sample preparation.
(2) Western blotting for DARTS verification: The cell lysate was individually treated with 0.5% DMSO, 0.8, 4, and 20 μM Bap on ice for 30 min. Next, 0.1% protease (at a ratio of protease concentration to sample protein concentration) was added to each group, which was then incubated at room temperature for 20 min.

### 2.10 Total glutathione peroxidase activity assay

The cells were cultured and treated with 0.5% DMSO, 0.8, 4 and 20 μM Bap with three biological replicates in 6- well plate for 24 h. The protein concentration of the cell lysate was determined by the Pierce™ BCA Protein Assay Kit (Thermo Scientific). The total glutathione peroxidase activity was analyzed using the Total Glutathione Peroxidase Assay Kit with NADPH (S0058, Beyotime Biotechnology, China), according to the manufacturer's instructions. The protein concentration was determined by the Pierce™ BCA Protein Assay Kit (Thermo Scientific). Relative quantification of the total glutathione peroxidase activity was performed based on the protein amount in each sample.

### 2.11 Assay of superoxide dismutase activity

The cells were cultured and treated with 0.5% DMSO, 0.8, 4 and 20 μM Bap with three biological replicates in 6- well plate for 24 h. The protein concentration of the cell lysate was determined by the Pierce™ BCA Protein Assay Kit (Thermo Scientific). The activity of superoxidase dismutase in each sample was assayed by using the Total Superoxide Dismutase Assay Kit with WST - 8 (S0101S, Beyotime Biotechnology, China), according to the manufacturer's instructions. Relative quantification of the superoxide dismutase activity was performed based on the protein amount in each sample.

### 2.12 Superoxide dismutase 3 (SOD3) activity assay

Diluted the purchased SOD3 active protein (638B4897DE, CLOUD-CLONE CORP., China) with PBS to 0.1 mg/mL. Took 65 μL SOD3 protein solution to four 1.5 mL EP tubes and added Bap (final concentrations of 0, 0.8, 4, and 20 μM) respectively. Incubated on ice for 10 min and tested the activity of SOD3 according to the instructions of the Total Superoxide Dismutase Assay Kit with WST - 8 (S0056, Beyotime Biotechnology, China).

### 2.13 Glutathione peroxidase 4 (GPX4) activity assay

Diluted the purchased GPX4 active protein (5F0F120B8F, CLOUD-CLONE CORP., China) with PBS to 0.1 mg/mL. Took 65 μL GPX4 protein solution to four 1.5 mL EP tubes and added Bap (final concentrations of 0, 0.8, 4, and 20 μM) respectively. Incubated on ice for 10 min and tested the activity of GPX4 according to the instructions of the Total Glutathione Peroxidase Assay Kit with NADPH (S0056, Beyotime Biotechnology, China).

### 2.14 Molecular docking

Molecular docking is a technique used to predict and simulate the interaction between receptors and ligands. The calculation formula of binding energy is as follows:

$$E=-R\times T\times \log\;K_{eq}$$

which R, T, and Keq stand for the ideal gas constant, Kelvin temperature, and equilibrium constant in the binding process, respectively. In this study, the protein structures of GPX4 (PDB ID: 7L8Q) and SOD3 (PDB ID: 2JLP) were obtained from the RCSB PDB database (https://www.rcsb.org/). The affinity between Bap or heparin with the target proteins was analyzed through Autodock Vina. The poses and vacuum electrostatic surfaces of the ligands or proteins were presented using Pymol (https://pymol.org).

### 2.15 ITC assay

Determined the binding models between Bap and GPX4 or SOD3 (Dingke Biotechnology Co., Ltd, China) by using the Nano ITC instrument (Microcal Inc., Northampton, MA, USA). Bap solutions (50 μL, 0.8 mM) in the syringe were titrated into the sample cell filled with GPX4 or SOD3 (250 μL, 0.05 mM) with 250 s intervals between each injection, the temperature was maintained at 25 °C.

### 2.16 Transfection of GPX4, SOD3 expressed plasmid

To explore the role of GPX4 and SDO3 in Bap-induced oxidative damage, the mouse GPX4 sequence (GenBank Accession No: NC_000076) and the mouse SOD3 sequence (GenBank Accession No: NC_000071) containing the open reading frame were cloned into the pCMV- 3 × HA- MCS- Neo vector to generate GPX4 overexpression plasmid and SOD3 overexpression plasmid. Cells transfected with these plasmids, as well as cells transfected with the vector plasmid, were designated as GPX4-overexpression (GPX4-OE), SOD3-overexpression (SOD3-OE), and vector, respectively. The constructed plasmids were transfected into cells using Lipofectamine 3000 reagent. Transfection efficiency was confirmed by western blotting (Figs. S1B and C).

### 2.17 Cell viability

The AML12 cells were seeded in 96 -well plates at a density of 5 × 10^4^ cells mL^−1^ and maintained at 37 °C with 5% CO_2_ in Dulbecco's Modified Eagle Medium containing 10% fetal bovine serum (FBS), 2 mM l-glutamine and 1% pyruvate. Cell viability was detected using Cell Counting Kit −8 Reagents (Thermo Scientific, China) after incubation with 4 μM Bap (dissolved in 0.3% DMSO) for 24 h, and reading the absorbance at 450 nm according to the product instruction.

### 2.18 The cells imaging of lipid ROS

Cells were seeded at a density of 2.5 × 10^5^ per well on coverslips placed in a 6- well dish for overnight. After 24 h of Bap exposure, cells were washed twice in PBS and incubated in a serum-free medium containing 2 μM C11- BODIPY 581/591 fluorescence probe (Invitrogen) added to per well, and incubated at 37 °C with 5% CO_2_ for 20 min. Coverslips were then washed twice with PBS and inverted onto microscope slides. Slides were imaged using a Nikon ECLIPSE 50i microscope.

### 2.19 Statistical analysis

Data were analyzed by one-way ANOVA and Duncan's test, using R software except for mass spectrometry analysis. Data are presented as the mean ± SD, repeat at least three biological samples per group. Significance was set at *P* < 0.05 and expressed as * *P* < 0.05, **P < 0.01, and ***P < 0.001.

## 3 Results

### 3.1 The toxicity of Bap on AML12 cells

The cell viability was assessed by treating cells with various concentrations of Bap for 24 h. The results indicated a dose-dependent decrease in cell viability (from 0.0064 to 500 μM, mean ± SD) upon Bap treatment (Fig. S1 A).

### 3.2 Bap induced metabolic reprogramming in AML12 cells

**Fig. 2:**
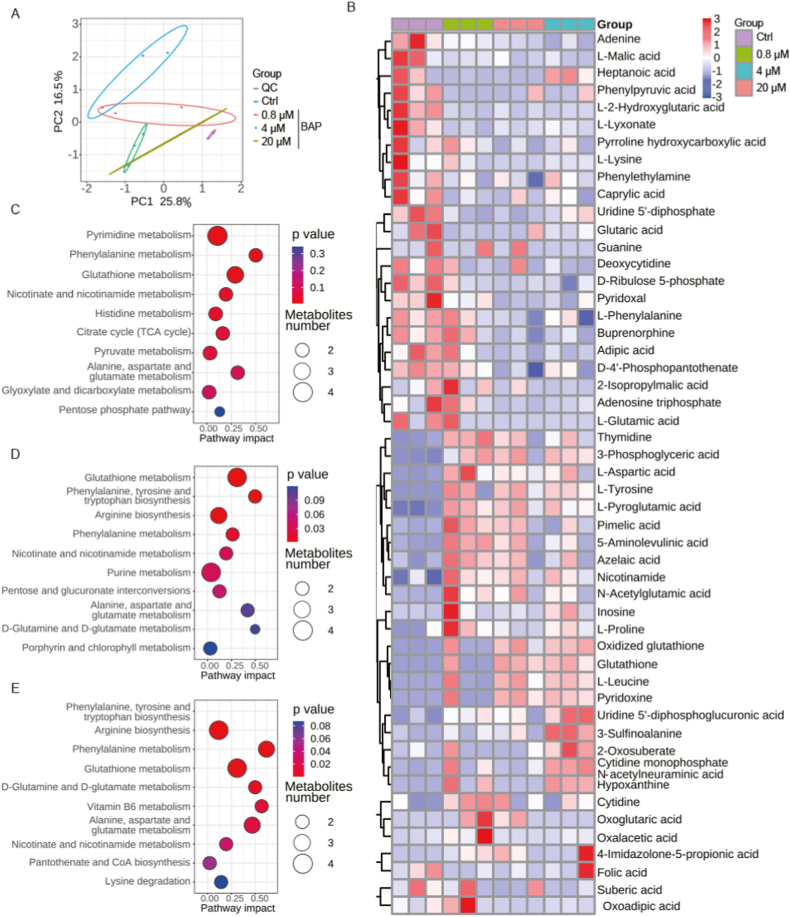
Metabonomic analysis of Bap exposure in AML12 cells. (A) PCA score map of the m/z - RT characteristics detected in the QC samples, dimethyl sulfoxide and 0.8, 4, and 20 μM Bap treatment group. (B) Thermogram and cluster analysis of changes in metabolite abundance in the DMSO, 0.8, 4, and 20 μM Bap treatment groups. Significant changes in metabolite KEGG pathway enrichment analysis in the 0.8 μM (C), 4 μM (D) and 20 μM (E) Bap treatment groups.

To mimic Bap exposure in the human body as closely as possible [5,26,32], doses of 0.8, 4, and 20 μM were chosen for the metabolomics analysis. The metabolic changes before and after exposure to Bap were analyzed using metabolomics. As shown in Fig. 2A, the high degree of clustering of quality control (QC) samples indicated good reproducibility for the metabolomics analysis. Additionally, the principal component analysis (PCA) obtained from the negative ion model demonstrated significant differences in metabolites between the control group and the treatment groups at different doses. The heatmap of the significantly changed metabolites indicated that Bap exposure resulted in a REDOX homeostasis imbalance in cells, with some changes exhibiting dose-dependent effects (Fig. 2B). Notably, oxidized glutathione, glutathione, and nicotinamide exhibited multiple changes and increased significantly upon Bap exposure. Furthermore, KEGG pathway analysis revealed that Bap primarily affected glutathione metabolism in AML12 cells (Fig. 2C–E).

### 3.3 Bap caused AML12 oxidative damage

**Fig. 3:**
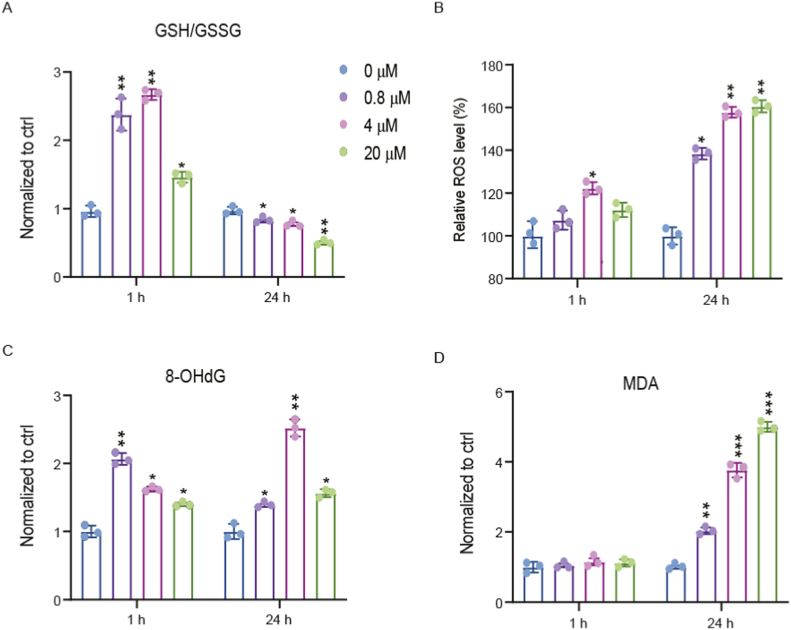
Bap destroyed the oxidative homeostasis of AML 12 cells. (A) The ratio of GSH and GSSG after 1 h and 24 h of 0.8, 4, and 20 μM Bap exposure. (B) Intracellular ROS levels in the DMSO, 0.8, 4, and 20 μM Bap treated groups. (C) Intracellular 8-OHDG levels in the Bap DMSO, 0.8, 4, and 20 μM treated group. (D) Intracellular malondialdehyde levels in the DMSO, 0.8, 4, and 20 μM Bap treated groups. Data are represented by box plots (n = 3 independent experiments). *P < 0.05, **P < 0.01, ***P < 0.005 compared with the vehicle control.

According to metabolomics analysis, Bap was found to affect the glutathione metabolism pathway, but it was unclear whether this effect was harmful. Therefore, we measured intracellular ratio of GSH and GSSG through LC-MS after 1 h and 24 h of Bap exposure. Our findings revealed that after 1 h of Bap intervention, GSH/GSSG significantly increased (Fig. 3A) due to activation of the cellular antioxidant system [[33], [34], [35]]. However, after 24 h of Bap exposure, GSH/GSSG significantly decreased (Fig. 3A), suggesting that Bap exposure ultimately disrupted the antioxidant system of AML12. As is well known, the decrease of GSH/GSSG indicates an increase in cellular oxidative damage [36,37]. Therefore, we hypothesized that Bap may cause oxidative damage to cells by interfering with glutathione metabolism. To further validate the hypothesis, we measured the levels of Bap-induced oxidative emergency damage markers. Consistent with previous studies [[38], [39], [40]], we found that Bap exposure significantly increased ROS (Fig. 3B), 8-OHDG (Fig. 3C) and MDA (Fig. 3D) levels in AML12 cells, these findings confirmed that Bap exposure can lead to oxidative damage in cells.

### 3.4 Screening Bap binding proteins with CETSA

**Fig. 4:**
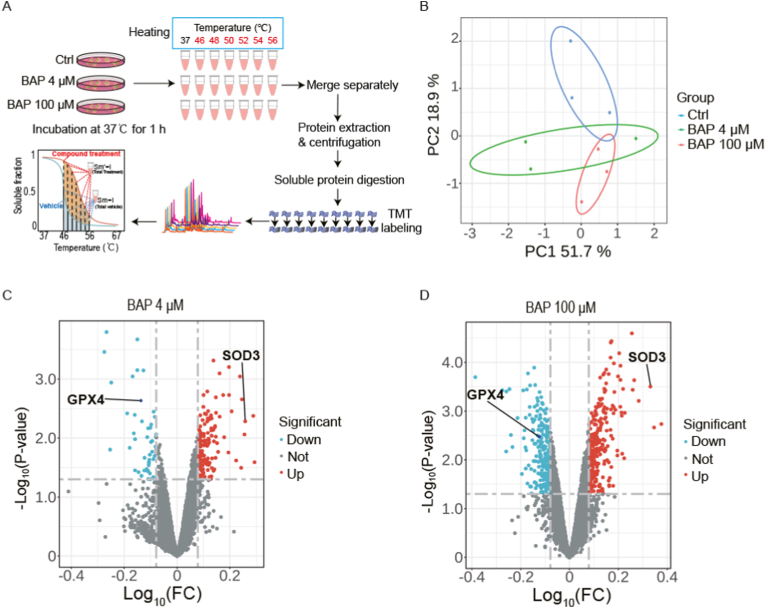
CETSA analyzed possible targets of Bap. (A) CETSA is applied to the workflow of screening Bap target proteins in cells. (B) PCA score map of m/z- RT features detected by the DMSO and 4 and 100 μM Bap treatment groups. (C) In the electric spray positive ionization mode, comparison of volcanic maps between the 4 μM Bap treatment group and the vehicle group. (D) In the electric spray positive ionization mode, a comparison of volcanic maps between the 100 μM Bap treatment group and the vehicle group.

In label-free chemical proteomics, in order to find the competitive targets, the low molecular weight compounds (<300 Da) typically require higher concentration (>100 μM) target protein screening processing [41]. Thus, to capture the impactful changes in the chemical proteome, 4 and 100 μM Bap were used for the target identification. In order to comprehensively explore the mechanism of Bap induced oxidative damage in cells, CETSA technology was utilized to screen the target proteins of Bap. CETSA relies on the principle that ligand binding results in thermal stabilization (or destabilization) of the target protein, the detailed workflow is shown in Fig. 4A. Initially, cells were subjected to Bap or DMSO intervention and heated at 37, 46, 48, 50, 52, 54, and 56 °C, respectively. Subsequently, the extracted proteins were labeled with TMT tags for quantitative analysis. During data processing, the quantitative data was normalized with the DMSO control group, proteins with significant enrichment factor changes bigger than 1.2 in the Bap treatment group were selected as potential targets. The PCA results showed significant changes in Bap binding protein in the supernatant (Fig. 4B). Specific analysis results indicated that there were 131 proteins in the 4 and 100 μM Bap treatment group identified as potential targets (Table S2). Among them, GPX4 and SOD3, which mainly regulate REDOX metabolism of AML12 cells, were identified with high confidence, with the fold changes of 1.4/1.4 and 1.8/2.1 in the 4/100 μM Bap treatment groups (Fig. 4C and D).

### 3.5 Binding affinity evaluation between Bap and the potential target proteins

**Fig. 5:**
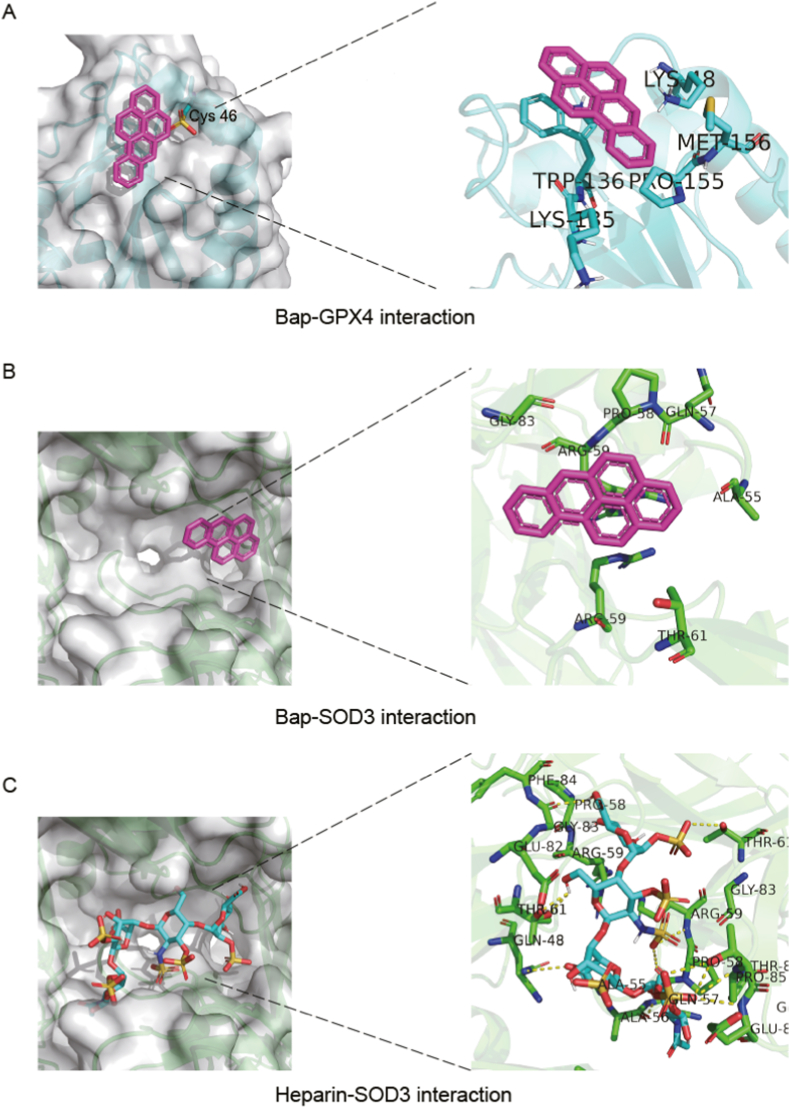
Docking analysis of GPX4 and SOD3 with Bap. The docking sites are shown as (A) Bap and GPX4; (B) Bap and SOD3; (C) Heparin and SOD3. The docking slots for GPX4, Heparin and SOD3 are (58, 60, 58, 0.064, - 3.409 and 7.043), (31, 31, 31, 22.459, - 11.035 and 30.44) and (31, 31, 31, 22.459, - 11.035 and 30.44), respectively.

To investigate the affinities between Bap and the target proteins (GPX4 and SOD3), simulation predictions were conducted by using molecular docking analysis. Fig. 5A depicts the surface of GPX4 (PDB ID: 7L8Q), which appears relatively flat and lacks of a well-defined binding pocket, but Bap is capable of covering the selenocysteine active site at position 46 on the protein's surface. Numerous studies have demonstrated that selenocysteine at position 46 is crucial for GPX4 activity, and many GPX4 inhibitors exert their effects through covalent binding with this specific site [[42], [43], [44], [45]]. From the molecular docking diagram, Bap was surrounded by hydrophobic residues on the binding side of the selenocysteine, and has strong electrostatic and van der Waals forces. The binding energy of Bap and GPX4 was calculated to be −7.8 kcal/mol, indicating a high binding ability between Bap and GPX4. Furthermore, Fig. 5B demonstrated that Bap could be embedded into the activity binding pocket of SOD3 (PDB ID: 2JLP). From the molecular docking diagram, Bap is also surrounded by hydrophobic residues on the binding side of the selenocysteine. The binding energy of Bap with SOD3 was calculated as - 9 kcal/mol. However, previous studies pointed out that the binding energy of heparin (which is closely related to SOD3 mediated antioxidant effect) and SOD3 was – 7.2 kcal/mol, and the affinity was much lower than Bap and SOD3 (Fig. 5C). In summary, both the docking simulation and the docking binding energy demonstrated the excellent binding abilities of Bap to SOD3 and GPX4.

### 3.6 Binding thermodynamic constants between Bap and the target proteins

**Fig. 6:**
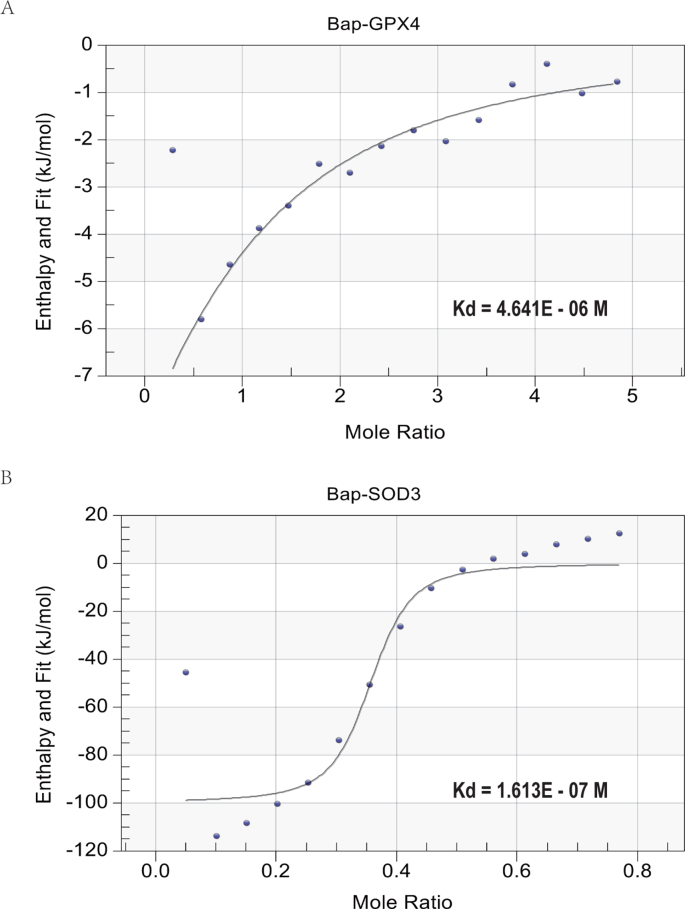
(A) Nano ITC assay for the thermal variation when Bap bound with GPX4; (B) Nano ITC assay for the thermal variation when Bap bound with SOD3.

To evaluate the binding thermodynamic constants between Bap and the target proteins, ITC, a physical technique for studying ligand receptor binding by determining thermodynamic parameters of interactions in solutions, was employed in this study [46]. The power vs. time titration curve obtained from the nano ITC test showed that there was a strong reaction between Bap and GPX4 or SOD3. In the reaction between Bap and GPX4 “Δ H < 0, Δ S > 0” is a slight exothermic process with significant entropy increase, and the dissociation equilibrium constant Kd was calculated as 4.46 μM (Fig. 6A). In the reaction between Bap and SOD3 “Δ H < 0, Δ S < 0” is a process of exothermic and significant entropy reduction, and the dissociation equilibrium constant Kd was calculated as 0.161 μM (Fig. 6B). The specific values are shown in Table S3 and Table S4. At a certain temperature and pressure, both types of combined Δ G values are negative, indicating that both types of binding processes can occur spontaneously.

### 3.7 Bap reduced enzyme activities of SOD3 and GPX4

**Fig. 7:**
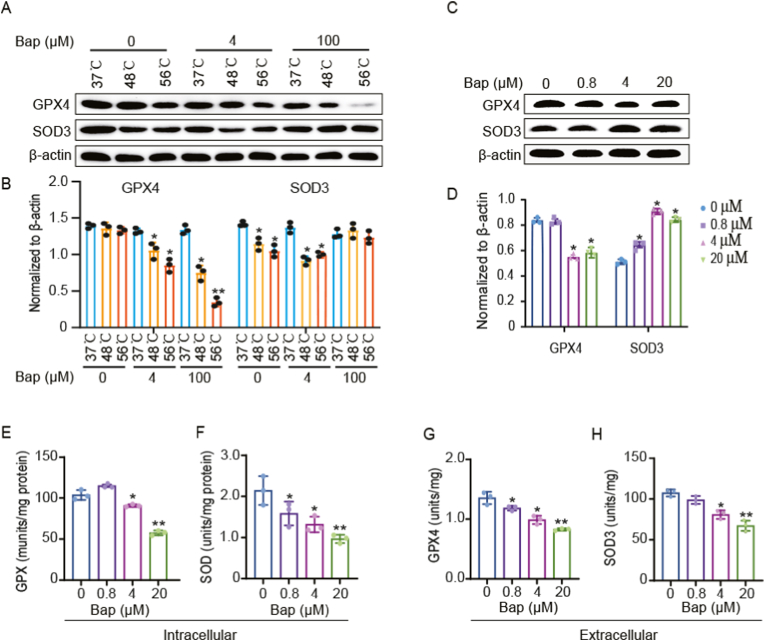
Bap inhibited GPX4 and SOD3 enzyme activity. (A) The protein extracted from AML12 cell lysate was used for CETSA *in vitro*. The indicated protein specificity was analyzed by western blotting using antibodies. (B) Densitometric analysis was performed as described in Materials and Methods. (C) The protein extracted from AML12 cell lysate was used for DARTS *in vitro*. The indicated protein specificity was analyzed by western blotting using antibodies. (D) Densitometric analysis was performed as described in Materials and Methods. (E) Detection of intracellular glutathione peroxidase activity in the DMSO, 0.8, 4, and 20 μM Bap treated group. (F) Detection of intracellular superoxide dismutase activity in the DMSO, 0.8, 4, and 20 μM Bap treated groups. (G) Detection of glutathione peroxidase activity after incubation with DMSO, 0.8, 4, and 20 μM Bap. (H) Detection of superoxide dismutase activity after incubation with DMSO, 0.8, 4, and 20 μM Bap. Data are represented by box plots (n = 3 independent experiments). *P < 0.05, **P < 0.01 compared with vehicle control.

To validate that Bap directly binds to GPX4 and SOD3 rather than its metabolites, the extracellular extracted cell proteins were incubated with Bap by additional CETSA and DARTS experiments. The results showed that Bap reduced the thermal stability of GPX4 and increased the thermal stability of SOD3 in a dose-dependent manner (Fig. 7A and B). Meanwhile, Bap enhanced the susceptibility of GPX4 to protease hydrolysis and increased the resistance of SOD3 to protease hydrolysis (Fig. 7C and D). And, Bap treatments significantly reduced the activity of GPX and SOD enzymes in cells (Fig. 7E and F). To further confirm that Bap directly inhibited the activity of SOD3 and GPX4 proteases, rather than indirectly effects. We detected enzyme activity by incubating the purchased active proteins of SOD3 and GPX4 with Bap for 10 min. The results showed that Bap dose-dependently inhibited the activity of SOD3 and GPX4 (Fig. 7G and H). In addition, even after 1 h or 24 h of Bap exposure, a substantial concentration of Bap remained in the cells, despite some metabolization into Benzo[a]pyrene 7,8-diol (Fig. S2). This provided strong evidence for the direct involvement of Bap in affecting the activity of SOD3 and GPX4 enzymes. Overall, our data collectively elucidated the potential toxicity of Bap in inhibiting GPX4 and SOD3.

### 3.8 Overexpression of GPX4 or SOD3 inhibited Bap-induced oxidative damage

**Fig. 8:**
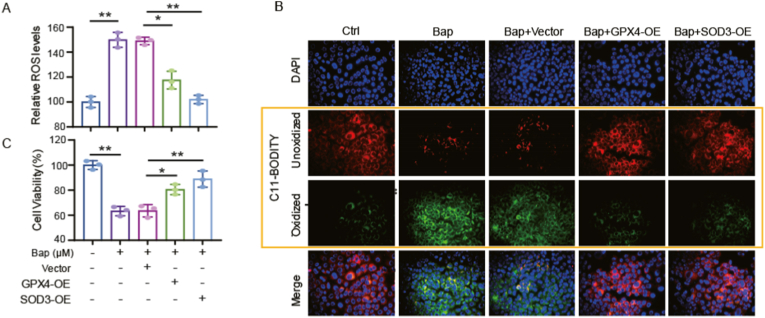
Overexpression of GPX4 or SOD3 alleviates Bap-induced oxidative damage. AML12 cells, GPX4 overexpressing cells or SOD3 overexpressing cells were treated with DMSO or 4 μM Bap for 24 h, then (A) the detection of ROS levels in cells by DCFHDA; (B) lipid peroxidation was measured using C11- BODIPY 581/591. Oxidized BODIPY C11 (Green) and unoxidized BODIPY C11 (Red) were imaged by fluorescent microscope. Scale bars: 50 μm; (C) Cells survival rate detected by CCK8. Data are expressed as the mean ± SD. *P < 0.05, **P < 0.01 compared with vehicle control. (For interpretation of the references to colour in this figure legend, the reader is referred to the Web version of this article.)

GPX4 is a selenium-containing phospholipid peroxidase, which protects cells from iron death caused by lipid peroxidation by reducing peroxidized lipids [[47], [48], [49]]. SOD3 is an extracellular enzyme that regulates cell redox conditions by catalyzing the disproportionation of superoxide to hydrogen peroxide [[50], [51], [52]]. In order to clarify whether Bap induces oxidative stress damage of cells through the mediation of GPX4 or SOD3, we conducted experiments to inhibit the oxidative toxicity of Bap by transiently transfecting cells with GPX4 or SOD3 overexpression. In the present study, the overexpression of both GPX4 and SOD3 was found to alleviate the oxidative damage induced by Bap. As shown in Fig. 8, the overexpression of GPX4 or SOD3 reduced the levels of ROS (Fig. 8A) and lipid peroxidation (Fig. 8B). Moreover, the cell viability assay supported that Bap caused oxidative damage via overexpression of GPX4 and SOD3 in AML12 cells (Fig. 8C).

## 4 Discussion

In summary, a comprehensive analysis was conducted on the metabolic product changes induced by Bap in hepatocytes. The findings suggested that Bap disrupted the redox homeostasis, leading to oxidative stress damage. The integration of metabolomics and chemical proteomics enabled the speculation that Bap inhibits glutathione metabolism, which subsequently affects the antioxidant effect of glutathione. In addition, the study evaluated the binding affinity between Bap and GPX4 or SOD3 through docking analysis and confirmed the interaction through ITC. These results addressed the knowledge gap regarding how Bap induces oxidative stress damage, rather than its metabolite. While metabolomics provides phenotype and function-related pathways, it cannot directly identify the upstream protein targets [53]. In contrast, chemical proteomics offers a complete list of proteins that the chemical can bind with, but it does not determine the associated bioactivities [54]. The integration of metabolomics and chemical proteomics enabled accurate mapping of the “top-down” mechanism within the living cell system. Overall, the methods used in this study provided valuable insights into the mechanisms underlying Bap-induced hepatotoxicity and may guide the development of preventive measures and therapeutic strategies.

Based on the above data, we believe that Bap, as a widespread polycyclic aromatic hydrocarbon pollutant, not only has its metabolites mutagenicity and carcinogenicity [55], but it also disrupts the redox homeostasis. This article proposed for the first time that Bap inhibited its antioxidant activity by binding to SOD3 and GPX4 through a combination of metabolomics and CETSA, rather than its metabolites. This study not only provided a new understanding of the mechanisms of Bap induced hepatotoxicity, but also provided a practical method for identifying the targets and mutual mechanisms of various environmental compounds.

However, it should be noted that this study had certain limitations that need further confirmation and investigation. Despite our findings suggested that GPX4 was targeted by Bap, the binding efficiency of GPX4 is hindered by the small size of the protein and the unique characteristics of its smooth surface. In addition, various studies have shown that SOD3 can bind protein glycans and extracellular matrix components on the cell surface through the positively charged C-terminal region [51,[56], [57], [58]]. Based on these characteristics, SOD3 is considered an effective antioxidant in extracellular space, and protects cells and biological molecules from superoxide-induced damage [[59], [60], [61], [62]]. Of course, literature also pointed out that SOD3 is also localized in the intracellular compartments of neutrophils and macrophages [50,63,64]. Although this provided a reasonable explanation for the binding of Bap to SOD3 detected in cell lysate, it cannot whether the SOD3 bound by Bap is a mature SOD3. Therefore, further exploration of the relationship between Bap and SOD3 is of great significance in explaining the oxidative damage toxicity of Bap.

In addition, we proposed a method to explain the intrinsic mechanism of toxic effects from environmental exposure, which involves determining the corresponding action pathway through phenotypic changes and utilizing a label-free chemical proteomics approach to screen for direct targets. This method could broaden the scope of applicability to a diverse array of environmental compounds. Especially in the compound that is inconvenient for modification or screening a large number of compound databases. Through our investigations, we have also concluded that the combination of metabolomics and CETSA holds significant promise in investigating complex systems, such as those involving target groups affected by the mixture effects or cocktail drugs of environmental compounds.

## Formatting of funding sources

Funding: This work was supported by the 10.13039/501100001809National Natural Science Foundation of China [grant number 82204584]; Chinese Pharmacopoeia Commission [Grant No.: 2022Y12]; and the Fundamental Research Funds from 10.13039/501100010877Shenzhen Science and Technology Innovation Commission [Grant No. RCBS20210609104424065].

## CRediT authorship contribution statement

**Yanwei Wang:** Methodology, Investigation, Writing -Original draft, Data curation. **Jiahui Zhao:** Investigation, Visualization. **Yipeng Xu:** Investigation, Writing-Review & Editing. **Cimin Tao:** Investigation, Data analysis. **Jie Tong:** Investigation, Writing-Review & Editing. **Yingjie Luo:** Writing -Review & Editing, Funding acquisition. **Yong Chen:** Investigation, Writing -Review & Editing. **Xuesong Liu:** Funding acquisition. **Tengfei Xu:** Conceptualization, Writing-Review & Editing, Project administration, Funding acquisition.

## Declaration of competing interest

The authors declare that they have no known competing financial interests or personal relationships that could have appeared to influence the work reported in this paper.

## Data Availability

Data will be made available on request.

## Appendix A Supplementary data

Multimedia component 3 Multimedia component 3

Fig. S1 Bap dose screening and validation of GPX4 and SOD3 overexpression plasmids. AML12 cells were treated with DMSO or Bap of concentration gradient for 24 h, then (A) cells survival rate detected by CCK8. (B) The GPX4 overexpression plasmid was transfected into AML12 cells for 72 h, and the levels of GPX4 protein were detected by Western blotting. (C) The SOD3 overexpression plasmid was transfected into AML12 cells for 72 h, and the levels of SOD3 protein were detected by western blotting. Data are expressed as the mean ± SD. **P* < 0.05, ***P* < 0.01 compared with vehicle control. Fig. S1

Fig. S2 LC-MS detection of intracellular Bap and its metabolite Benzo [a] pyrene7,8-diol concentration after Bap exposure. AML12 cells were treated with DMSO, 0.8, 4, and 20 μM Bap for 1 h or 24 h. Then, detected Bap and its metabolite Benzo [a] pyrene 7,8 - diol concentration in AML12 cells by LC-MS. Data are expressed as the mean ± SD. **P* < 0.05, ***P* < 0.01, ****P* < 0.005 compared with vehicle control. Fig. S2
