# Supplementary material for: Uncovering SOD3 and GPX4 as new targets of Benzo[α]pyrene-induced hepatotoxicity through Metabolomics and Chemical Proteomics
Source: Redox Biol. 2023 Oct 11;67:102930. doi: 10.1016/j.redox.2023.102930 (PMC10585396; doi:10.1016/j.redox.2023.102930)
Supplement: Multimedia component 1 [file mmc1.docx]

**Supporting information**

**Uncovering SOD3 and GPX4 as new targets of Benzo[α]pyrene-induced hepatotoxicity through Metabolomics and Chemical Proteomics**

This supporting information includes two Texts and two Tables

Text S1. Metabolite extraction, profiling, and identification………………………………… 2

Text S2. Proteome sample analysis and quantitation………………………………………….3

Table S3. Thermodynamic parameters of Bap bounding with GPX4…………………………4

Table S4. Thermodynamic parameters of Bap bounding with SOD3…………………………4

Text S1. Metabolite extraction, profiling, and identification

The collected cells were homogenized in 0.25 M Tris sucrose EDTA (pH 7.4) and 0.1% sodium dodecyl sulfate, and then homogenized with a solution containing water, methanol, and chloroform (1:1.5:2 (v/v). The organic phase was dried under N2 and resuspended in 0.5mL of methanol. Subsequently, through the Acrodisc filter (0.45 μ m. 25mm diameter, Gelman Sciences, Ann Arbor, MI, USA) removes particles from the extract. Finally, the extract was stored in dark at 4 degrees Celsius until analyzed. The UHPLC- Q- Exactive Orbitrap MS analysis was conducted using a ThermoFisher Scientific Vanquish Ultra- High Performance Liquid Chromatography system and a ThermoFisher Scientific Q- Exactive Orbitrap mass spectrometer. The samples were separated on a UPLC BEH amide column (1.7 µm, 100 mm × 2.1 mm; Waters, Milford, MA, USA). In the negative ion mode, the mobile phase consisted of eluent A (water containing 25 mM NH_4_OH and 25 mM NH_4_OAc) and eluent B (acetonitrile). The settings for the linear gradient were as follows: 0 to 0.5 min: 95% B; 0.5 to 9 min: 95% B to 65% B; 9 to 10 min: 65% B to 40% B; 10 to 12 min: 40% B; 12 to 12.1 min: 40% B to 95% B; 12.1 to 15 min: 95% B, with flow rate 0.4 mL/ min. In the positive ion mode, the mobile phase consisted of eluent A (water containing 0.1% formic acid) and eluent B (acetonitrile), the settings for the linear gradient were as follows: 0 to 1 min: 5% B; 1 to 10 min: 5% B to 100% B; 10 to 12 min: 100% B; 12 to 12.1 min: 100% B to 5% B; 12.1 to 15 min: 5% B, with flow rate 0.4 mL/ min. Mass detection was carried out in both positive and negative modes, with the ion spray voltage set at 4.5 and- 3.0 kV, respectively. The interface temperature, heat block temperature, and desolvation line temperature was set at 300°C, 400°C, and 250°C, respectively. The flow rate of the drying and nebulizing gas was set to 10 L/ min and 3 L/ min, respectively. The data were obtained in the scanning mode over a mass range of m/z 70 - 1050 Da in positive and negative ion models, with collision energies of 10, 20, and 40 eV for fragmentation. Metabolic data acquisition and pre-analysis were performed using Xcalibur Software v.4.1 (Thermo Fisher Scientific Inc.). The peaks of QC samples with a coefficient of variation (CV) >  30% were excluded before further processing. R was used for multivariate statistical analysis. The metabolites were identified by matching the MS/MS spectra in METLIN (http://metlin.scripps.edu/) and HMDB (http://www.hmdb.ca/). The metabolic pathways were analyzed based on the Kyoto Encyclopedia of Genes and Genomes (KEGG) database ([www.genome.jp/kegg/](http://www.genome.jp/kegg/)).

Text S2. Proteome sample analysis and quantitation.

The lysate was further digested to produce peptides for MS analysis. Briefly, a final concentration of 20 mM tris (2- carboxyethyl) phosphine (TCEP), 25% trifluoroethanol (TFE) (v/ v) and 35 µL 100 mM triethylammonium bicarbonate (TEAB) (pH 8.5) were added into a solution with 25 µL lysate sample, which was further incubated at 55℃ for 20 min. Then a final concentration of 55 mM 2- chloroacetamide (CAA) was added to the solution and incubated in dark for 30 mins for alkylation. Subsequently, 2.5 µg of trypsin was added to the solution and incubated overnight at 37℃. After digestion was completed, the samples were centrifuged at 20000 × g for 10 min. Dried the supernatant and resolubilized with 50 µL 100 mM TEAB (pH 8.5). TMT labels were added to each sample with amounts of 10 µL to 25 µg peptide and incubated the solution overnight at room temperature. The combined TMT labeled sample solution was desalinated using a C18 solid- phase extraction (SPE) column and further eluted on a C18 rotating column. Subsequently, the 2 μg peptide collected from each fraction was separated on a 50 cm × 75 μm Easy Spray column (Thermo Scientific) in a 90 mins gradient elution with mobile phase A (0.1% formic acid in water) and mobile phase B (0.1% formic acid in 99% acetonitrile) by the Proxeon 1000 UHPLC system coupled with an Orbitrap Exploris 480 mass spectrometer (Thermo Scientific). The raw MS data were acquired by data dependent acquisition at a resolution of 75,000 and 50,000 in MS and MS/MS, respectively. Identification and quantification of proteins using Proteome Discoverer 2.1 software (Thermo Scientific) and Mascot 2.6.0 (Matrix Science). The search parameters were set as follows: the precursor mass tolerance was set as 20 ppm, the fragment mass tolerance was set as 0.04 Da, the enzyme name was trypsin/P and the maximum missed cleavage site was set as 3. The dynamic modifications search parameters including acetylation (protein N- terminus), oxidation (M), deamidation (NQ), and the static modifications including carbamidomethylation (C) and TMT10 -plex (K and peptide N- terminus). Strict and relaxed target FDR were set as 0.01 and 0.05 in the decoy database search; respectively. The obtained peptide sequences were mapped by a local mouse proteome Uniprot library sequence entries. For the protein quantification, unique and razor peptides were used for the protein assignment and quantification, respectively. The reporter ions of the TMT channels were normalized and submitted for further statistical analysis. Ribosomal proteins (RPL) and S ribosomal proteins (RPS) were removed from the final list to get the target proteins with higher confidence.

Table S3. Thermodynamic parameters of Bap bounding with GPX4

| Model | Variable | Value |
| --- | --- | --- |
| Independent | Kd (M) | 4.64E -06 |
|  | n | 0.823 |
|  | ∆H(KJ/mol) | -10.06 |
|  | ∆G(KJ/mol) | -30.44 |
|  | ∆S(J/mol·K) | 68.35 |

Table S4. Thermodynamic parameters of Bap bounding with SOD3

| Model | Variable | Value |
| --- | --- | --- |
| Independent | Kd (M) | 1.613E -07 |
|  | n | 1.063 |
|  | ∆H(KJ/mol) | -100 |
|  | ∆G(KJ/mol) | -38.77 |
|  | ∆S(J/mol·K) | -205.4 |
