# Supplementary figures and images for: Uncovering SOD3 and GPX4 as new targets of Benzo[α]pyrene-induced hepatotoxicity through Metabolomics and Chemical Proteomics
Source: Redox Biol. 2023 Oct 11;67:102930. doi: 10.1016/j.redox.2023.102930 (PMC10585396; doi:10.1016/j.redox.2023.102930)

Figure S1

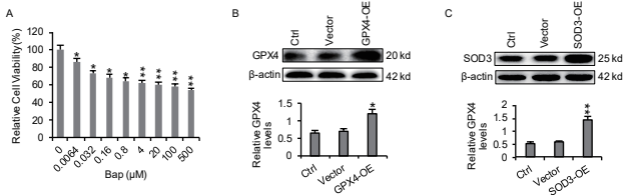

Supplement: Fig. S1 — Bap dose screening and validation of GPX4 and SOD3 overexpression plasmids. AML12 cells were treated with DMSO or Bap of concentration gradient for 24 h, then (A) cells survival rate detected by CCK8. (B) The GPX4 overexpression plasmid was transfected into AML12 cells for 72 h, and the levels of GPX4 protein were detected by Western blotting. (C) The SOD3 overexpression plasmid was transfected into AML12 cells for 72 h, and the levels of SOD3 protein were detected by western blotting. Data are expressed as the mean ± SD. *P < 0.05, **P < 0.01 compared with vehicle control. [file mmc4.pdf]

Figure S2

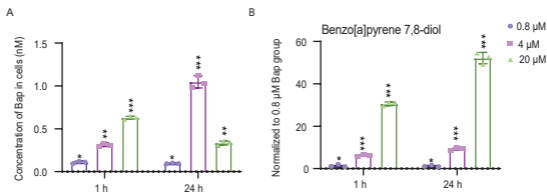

Supplement: Fig. S2 — LC-MS detection of intracellular Bap and its metabolite Benzo [a] pyrene7,8-diol concentration after Bap exposure. AML12 cells were treated with DMSO, 0.8, 4, and 20 μM Bap for 1 h or 24 h. Then, detected Bap and its metabolite Benzo [a] pyrene 7,8 - diol concentration in AML12 cells by LC-MS. Data are expressed as the mean ± SD. *P < 0.05, **P < 0.01, ***P < 0.005 compared with vehicle control. [file mmc5.pdf]
